# Supplementary material for: Deep Learning-Based Knee MRI Classification for Common Peroneal Nerve Palsy with Foot Drop
Source: Biomedicines. 2023 Nov 28;11(12):3171. doi: 10.3390/biomedicines11123171 (PMC10741167; doi:10.3390/biomedicines11123171)
Supplement: Supplementary file 1 [file biomedicines-11-03171-s001.zip › biomedicines-2695284-supplementary.pdf]

Figure S1. Representative images from knee magnetic resonance image with different sequence parameters.

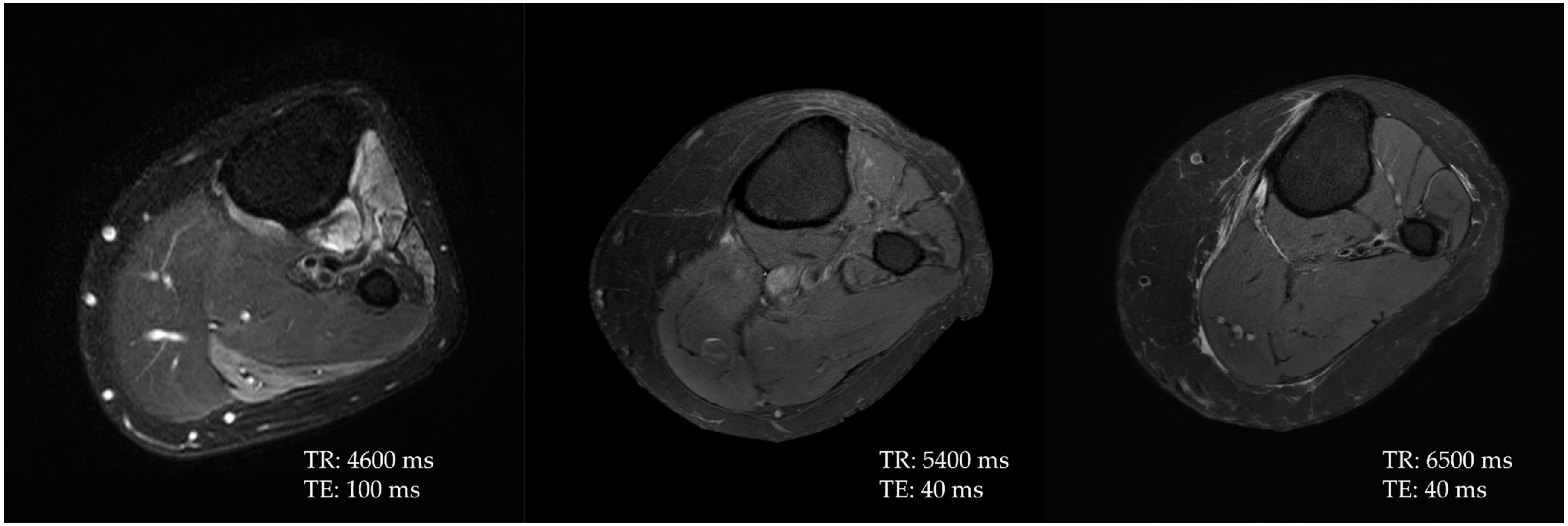

(a) **(b)** **(c)**

TR, repetition time; TE, echo time; ms, millisecond.

Figure S2. Schematic architectures of the VGG19, ResNet152, and EfficientNet-B5 algorithms.

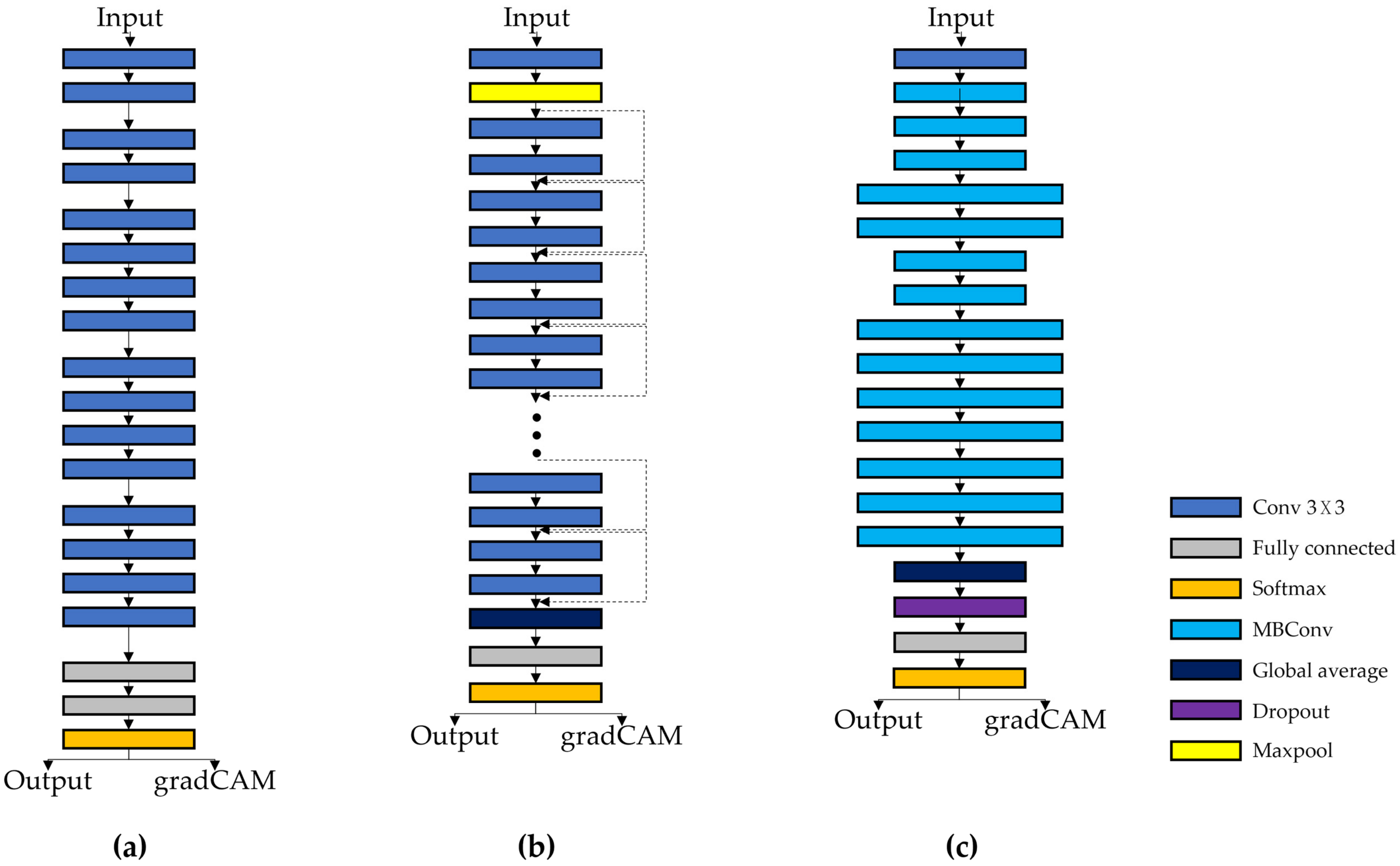

Boxes represent layers in a neural network. In the case of "conv 3 X 3", it represents a convolutional layer for segmentation purposes with a kernel size filter of 3 * 3. The gray box represents the fully connected layer, in which each neuron applies a linear transformation to the input vector through the weights matrix. Softmax layer (orange box) converts a vector of *K* real numbers into a probability distribution of *K* possible outcome (especially, binary in this article). MBConv (mobile inverted bottleneck convolution) layers creates an inverted residual block, which is characterized by a bottleneck structure that reduces the number of input channels while increasing the number of output channels. The global average pooling layer (indigo box) is the layer for making the features into a one-dimensional vector. The dropout layer (purple box) enables to prevent overfitting by randomly omitting some neurons. The Maxpool is a maximal polling layer that extracts largest values from the vectors of a given kernel size. In the figure, the arrows indicate the progression of the algorithm, and the dotted arrows indicate additional short-cut (skip) connections in ResNet, which serve to add input to the learned function.

Figure S3. The area under the receiver operating characteristics curves of deep learning models to predict the common peroneal nerve injury magnetic resonance image in the validation dataset.

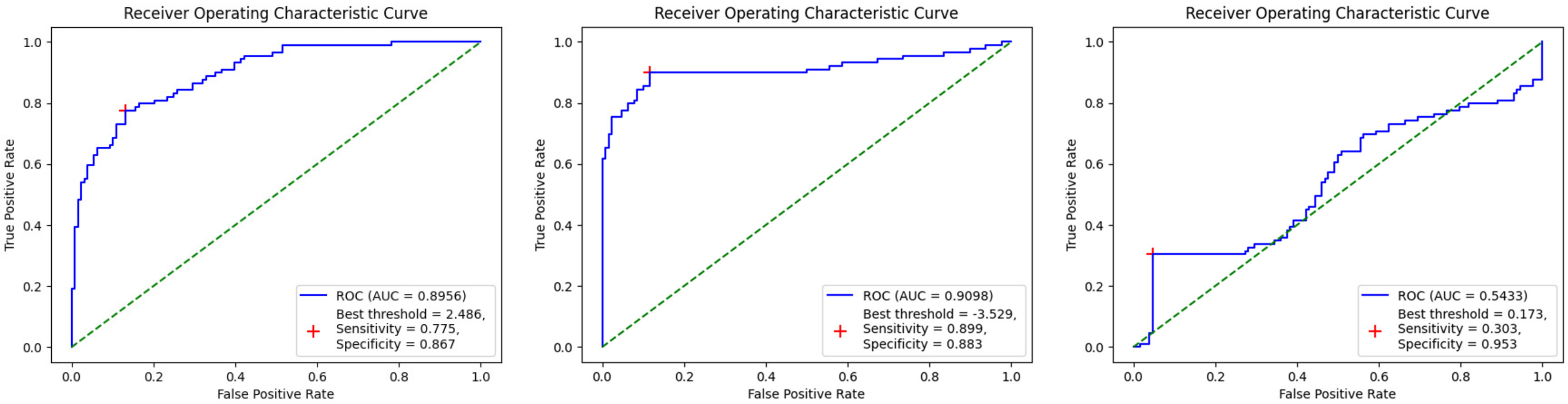

(a) **(b)** **(c)**

(a) the EfficientNet-B5, (b) ResNet152, and (c) VGG19.

Table S1. Optimized hyperparameters in each CNN-based model.

	VGG19	ResNet152	EfficientNet-B5
Batch size	16	16	4
Learning rate	10^{-2}	0.09	10^{-1}
Dropout	0.1	-	0.4
Epochs	20	100	150
